# Impact of Emerging, Re-Emerging and Zoonotic Viral Infectious Diseases, in a Virologist’s Perspective

**DOI:** 10.2174/1874357901812010131

**Published:** 2018-08-31

**Authors:** Nobumichi Kobayashi

**Affiliations:** Sapporo Medical University School of Medicine, Sapporo, Japan

Emerging and re-emerging viral infections have been a major threat to public health worldwide, since their recognition in the late 20th century [1]. These infectious diseases include those caused by newly identified viruses, previously known viruses that acquired additional virulence traits, and those showing spread to unaffected regions. In the last ten years, re-emergence has been noted for Zika, Ebola, MERS, Dengue, Chikungunya and avian influenza, while SFTS (severe fever with thrombocytopenia syndrome) was recognized to be caused by a novel virus. These diseases are free to move across national borders according to rapid human mobility via global airline network. With this background, any novel infectious disease anywhere in the world may have the potential for global spread.

Although various factors are considered to be associated with an increase of emerging/ re-emerging infectious diseases [2], they can be summarized as three major changes on the global level, *i.e*., (1) change in human/society/behavior, (2) change in environment/ecosystem, and (3) change in microorganisms. These changes are considered to synergistically increase the risks for the emergence of pathogens, transmission of pathogens, and opportunity of infection (susceptible hosts).

Factors related to humans and society are the most responsible for emergence and spread of infectious diseases. At present, global population is estimated to be 7.4 billion and growing rapidly, described as “a population explosion”. This increase is remarkable in sub-Saharan Africa and South Asia. Increase of population is a common issue in developing regions and facilitates poverty, and leads to urbanization associated with the growth of megacities (37 cities as of 2017) with a population of more than 10 million. High population and its density increase the risk for transmission of infectious pathogens *via* human-to-human contact, and low hygienic condition arising from undeveloped infrastructures (*e.g*., sewerage system). Naturally, population explosion and urbanization facilitate the expansion of residence area of humans, which promote forest development associated with environmental destruction. During the process, humans may encounter unknown viruses which have been lurking in any animal but harmful to humans. Although it has not yet been fully demonstrated, Ebola virus is suggested to reside in some species of fruit bats as potential natural host, from which this virus might have transmitted to humans, causing Ebola Virus Disease (EVD).

International tourists who traveled abroad reached over 1.1 billion in 2014, and have been constantly increasing. The development of airline networks enables infectious pathogen to spread globally in a few days. Such typical situation was evident for the outbreak of SARS (severe acute respiratory syndrome) in 2003, when the unknown pathogen, which was later revealed to be a novel coronavirus, was disseminated from China to at least 17 countries within a week via air travel of infected patients. The recent outbreak of MERS coronavirus in Korea (2015) was also caused by a returnee from the Middle East. Spread of emerging viruses is indirectly related to socioeconomic problems including civil wars, an increase of refugees, and natural disasters. These human factors always compromise human health and increase the risk for emerging/re-emerging infectious diseases.

More than 200 virus species have been known to be able to infect humans. Historically, the number of newly identified virus species/family has been constantly increasing since the 20th century, which was associated with the development of technology, from tissue culture and serological detection, to genetic identification represented by PCR and high throughput sequencing [3]. Among more than 1000 pathogens of humans, over 50% of them were considered to have originated in animal species (vertebrates). Emerging viruses are considered more likely to be zoonotic. The number of infectious diseases outbreaks increased globally about 4 times from the 1980s to 2010, associated with an evident increase of zoonosis as well as vector-borne disease, compared with human-specific infections [4]. Increase of zoonosis and vector-borne diseases is related to global changes in environment and ecosystem which may be caused by climate change associated with global warming. Floods, fierce heat, and drought which arise as an influence of climate variation, may cause an increase of vectors, facilitating their move from an endemic area, and a decrease of natural enemies to vectors. Phylogenetic analysis combined with chronological tracing indicated that recent global spread of Chikungunya was caused synergistically by factors of humans, environment, vectors, and viruses [5]. In the 1980s and 1990s, illegally dumped waste tires increased due to globalized trading and industrialization. These tires provided water pool for the proliferation of mosquitos, enhancing local endemicity. Thereafter, viruses with vectors have disseminated via international airline network, associated with the occurrence of genetic diversity in viral genome. A mutation in the envelope protein conferred increased viral growth in mosquito, which facilitated spread of this vector-born disease. Thus, spread of emerging viral diseases is considered to be caused by multifactorial mechanisms.

Currently, globally important viral infectious diseases may be classified into following categories with features such as (1) high frequency worldwide (diarrhea, respiratory infections, *etc*.), (2) pandemic concern (EVD, *etc*.), (3) directed to eradication/elimination (*e.g*., poliomyelitis), and (4) neglected tropical diseases (*e.g*., rabies). These viral diseases are prone to be prevalent in developing countries, because of 1) higher population (density), 2) higher prevalence of vector/reservoir of virus pathogens, 3) shortage of medical/preventive resources against viral diseases and 4) low socioeconomic/hygienic status. Furthermore, it is difficult to predict the emergence or spread of novel viral infections, and particularly zoonosis is impossible to eradicate because wild animals/vectors carry viral pathogens. Despite such situations, scientists have a significant role to reduce the risks of emerging viral diseases. The first priority is to ensure adequate surveillance of viral diseases, *i.e*., to maintain epidemiological study on humans, animals, virus strains from various sources including environment, which is relevant to “One Health” approach advocated recently. Molecular epidemiology and population dynamics of viruses provide suggestions for the genetic, phenotypic, and epidemiologic trend of the viral diseases. Outcomes from those efforts are definitely essential for developing diagnostic methods, therapeutic approaches, and vaccines.
